# Na_V_1.7 mRNA and Protein Expression in Resident Neurons of the Human Spinal Dorsal Horn

**DOI:** 10.1002/cne.70168

**Published:** 2026-05-15

**Authors:** Stephanie Shiers, Nwasinachi A. Ezeji, Carla Lima‐Truax, Jeffrey L. Krajewski, Geoffrey Funk, Anna Cervantes, Peter Horton, Gregory Dussor, Stephanie Hennen, Theodore J. Price

**Affiliations:** ^1^ Department of Neuroscience, Center for Advanced Pain Studies The University of Texas at Dallas Richardson Texas USA; ^2^ Eli Lilly and Company Indianapolis Indiana USA; ^3^ Southwest Transplant Alliance Dallas Texas USA; ^4^ Grünenthal Gmbh Aachen Germany

**Keywords:** dorsal horn, Na_V_1.7, spinal cord

## Abstract

The voltage‐gated sodium channel Na_V_1.7 is a pain target supported by human genetics, and many compounds have been developed to inhibit Na_V_1.7 but have disappointed in clinical trials due to cardiovascular effects. Because some recent reports suggest that pharmacological inhibition of Na_V_1.7 in the rodent spinal dorsal horn can achieve pain relief, we sought to better understand Na_V_1.7 expression in the human spinal cord. We report that Na_V_1.7 mRNA is expressed in putative projection neurons (NK1R+, GPR83+) in the human spinal dorsal horn, predominantly in lamina I and II, as well as in deep dorsal horn neurons. Na_V_1.7 mRNA was also detected in preganglionic parasympathetic and sympathetic neurons, motor neurons in the ventral horn, and ependymal cells lining the central canal. Na_V_1.7 protein was predominantly found in the central axons of sensory neurons terminating in lamina I–II and colocalized, in part, with presynaptic markers like Bassoon and CGRP. Postsynaptically, Na_V_1.7 protein was detectable in the soma of motor neurons but was more elusive in dorsal horn populations due to the abundance of presynaptic signal. However, Na_V_1.7 protein was detected in the axon initial segment of some resident dorsal horn neurons and in axons entering the anterior commissure. Given that projection neurons are critical for conveying nociceptive information from the dorsal horn to the brain, these data support that dorsal horn Na_V_1.7 expression may play an unappreciated role in pain phenotypes observed in humans with genetic *SCN9A* mutations.

## Graphical abstract

We identified Nav1.7 mRNA and protein in human spinal cord neurons, a subset of which co‐expressed putative projection neuron markers, *TACR1* and *GPR83*. These neurons relay nociceptive signals from the periphery to the brain. While Nav1.7 loss‐of‐function mutations cause congenital insensitivity to pain, traditionally linked to sensory neurons, our findings demonstrate Nav1.7 expression in spinal neurons, a feature not well‐supported in rodents. These results suggest that Nav1.7 may play an unappreciated role in pain through expression in spinal cord neurons.

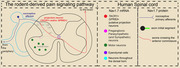

## 1 Introduction

Loss‐ and gain‐of‐function mutations in the gene encoding voltage‐gated sodium channel (VGSC) Na_V_1.7 (Baker and Nassar 2020), *SCN9A*, result in congenital pain insensitivity (Cox et al. 2006) or in extreme pain disorders like primary erythromelalgia (Mann et al. 2019; Yang et al. 2004), paroxysmal extreme pain disorder (Fertleman et al. 2006; Hua et al. 2022), and idiopathic small fiber neuropathy (Faber et al. 2012), respectively. Na_V_1.7 is predominantly expressed in the peripheral nervous system, including sympathetic neurons and nociceptive sensory neurons in the dorsal root ganglia (DRG) and trigeminal ganglia (Hameed 2019). It is localized to the membrane of sensory neurons innervating the skin, muscle, and other organs, and central axons that terminate in the dorsal horn of the spinal cord (Black et al. 2012; S. Shiers et al. 2020; S. I. Shiers et al. 2021). It is a key regulator of neuronal excitability as it mediates Na+ currents during membrane depolarization and action potential firing (McDermott et al. 2019; Middleton et al. 2022) and is upregulated in pain conditions in both rodents and humans (Alvarez et al. 2021; Black et al. 2008; Hameed 2019; Li et al. 2018; Liu et al. 2021; Nascimento et al. 2022; Sun et al. 2018). The peripheral nociceptive system has been considered as the primary site of action for therapeutic targeting of Nav1.7 (Chew et al. 2019). Several Na_V_1.7 inhibitors have been developed, and a series of clinical trials have been conducted with mixed reports on pain outcomes and cardiovascular safety (Alles and Smith 2021; Biogen 2021; Dormer et al. 2023; Eagles et al. 2022; Kingwell 2019; McDonnell et al. 2018; Price et al. 2017). While some of the lack of efficacy outcomes could be due to poor pharmacokinetics and/or limited selectivity toward other sodium channels (Kraus et al. 2021), recent studies demonstrate that inhibiting Nav1.7 on sympathetic neurons causes cardiovascular effects that reduce the safety profile of these direct inhibitors of Nav1.7 (Dib‐Hajj et al. 2013; Mulcahy et al. 2024).

While mostly absent in the rodent brain (Allen Institute for Brain Science 2022b; Lein et al. 2007) and human cortex (Allen Institute for Brain Science 2022a), Na_V_1.7 protein is localized to presynaptic axons in both the rodent and human spinal dorsal horn (Black et al. 2012; S. I. Shiers et al. 2021). Recent studies have suggested that Na_V_1.7 channels in the CNS are potentially important for analgesia. A central analgesic mechanism for Na_V_1.7 was recently proposed wherein Na_V_1.7‐null mice that were pain insensitive retained nociceptor firing properties but displayed opioid‐dependent impaired synaptic transmission and neurotransmitter release at sensory neuron presynaptic terminals in the spinal dorsal horn (MacDonald et al. 2021). There is also evidence for Na_V_1.7 expression and function postsynaptically within the spinal cord. For instance, a recent study demonstrated that dorsal horn neuron Nav1.7 expression is important for spinal cord injury pain (Fu et al. 2023). Na_V_1.7 mRNA has also been identified in several subsets of mouse spinal cord neurons using single‐cell RNA sequencing (Russ et al. 2021), including Phox2a+ projection neurons that convey pain, itch, and temperature information to the brain through the anterolateral system (ALS) (Bell et al. 2023). Another study using electron microscopy revealed that a substantial proportion of Na_V_1.7 immunoreactivity was localized to dendrites of mouse spinal dorsal horn neurons, but these authors proposed that the protein originated from presynaptic fibers and was transferred to postsynaptic sites through a mechanism that remains to be elucidated.

Given the evidence described above in the mouse, we hypothesized that *SCN9A* mRNA and Na_V_1.7 protein might be expressed by human dorsal horn neurons, including projection neurons. Here, we used a variety of techniques to present evidence of Na_V_1.7 mRNA and protein expression in a diverse subset of human dorsal horn neurons, including putative projection neurons. Our findings support that the functional role of spinal Nav1.7 in putative projection neurons should be investigated, as its expression in these cells may be important for human nociception.

## 2 Materials and Methods

### 2.1 Human Tissue Procurement

All human tissue procurement procedures were approved by the Institutional Review Boards (protocol Legacy‐MR‐15‐237) at the University of Texas at Dallas. All tissues were procured from neurologic determination of death organ donors through a collaboration with the Southwest Transplant Alliance (STA). The STA obtained informed consent for research tissue donation from the donor's family. Human spinal cords (lumbar to sacral levels) were recovered using a ventral approach (Valtcheva et al. 2016) from organ donors within 4 h after cross‐clamp, frozen immediately in powdered dry ice, and stored in a −80°C freezer. Details about the procurement process and tissue processing can be found on protocols.io (S. Shiers et al. 2024). Sex was not considered as a biological variable, as several studies have found no differences in Nav1.7 expression in DRG (Chang et al. 2018; Ghovanloo et al. 2024), and all experiments consist of pooled male/female data. Donor demographics and sample information are provided in Table 1.

**TABLE 1:** Donor demographics.

Donor ID	Age	Sex	COD	Spinal level	Experiment
UTD‐DN0042	44	F	CVA/stroke	Lumbar 4	SCN9A/TACR1
					SCN9A/GPR83
					Na_V_1.7/NeuN
					Na_V_1.7/MAP2
UTD‐DN0087	56	M	Head trauma/GSW	Lumbar 5/Sacral 1	SCN9A/TACR1
					SCN9A/GPR83
					Na_V_1.7/NeuN
					Na_V_1.7/MAP2
UTD‐DN0077	40	F	CVA/stroke	Lumbar 1	SCN9A/TACR1
					SCN9A/GPR83
					Na_V_1.7/NeuN
UTD‐DN0044	45	F	CVA/stroke	Lumbar 3	SCN9A/TACR1
					SCN9A/GPR83
					Na_V_1.7/NeuN
					Na_V_1.7/MAP2
UTD‐DN0023	18	F	Anoxia/seizure	Sacral 1	Na_V_1.7/CGRP/Bassoon
UTD‐DN0014	24	M	Head trauma/MVA	Lumbar 4	Na_V_1.7/CGRP/Bassoon
UTD‐DN0011	53	F	CVA/stroke	Lumbar 5/Sacral 1	Na_V_1.7/CGRP/Bassoon
UTD‐DN0037	69	M	CVA/stroke	Lumbar 5	Na_V_1.7/CGRP/Bassoon
UTD‐DN0091	22	M	Overdose/cardiovascular	Lumbar 5	Na_V_1.7/MAP2

### 2.2 RNAscope In Situ Hybridization

Frozen human spinal cords (∼1.5‐cm‐long segments) were gradually embedded in OCT in a cryomold by adding small volumes of OCT over dry ice to avoid tissue thawing. Human spinal cords were sectioned at 20 µm onto SuperFrost Plus charged slides (Fisher Scientific; Cat 12‐550‐15). Sections were only briefly thawed in order to adhere to the slide but were immediately returned to the −20°C cryostat chamber until the completion of sectioning. Three sections from each donor were stained in each experiment, and three to four donors were used in each experiment. The slides were removed from the cryostat, thawed at 37°C for exactly 1 min, and then immediately transferred to 10% formalin (Fisher Scientific; Cat 23–245684) for 15 min at room temperature. The sections were then sequentially dehydrated in 50% ethanol (5 min; Fisher Scientific; Cat 04‐355‐223), 70% ethanol (5 min), and twice in 100% ethanol (5 min each) at room temperature. The slides were air‐dried briefly, and then boundaries were drawn around each section using a hydrophobic pen (ImmEdge PAP pen; Vector Labs). Once the hydrophobic boundaries had dried, the slides were immediately processed for RNAscope in situ hybridization.

RNAscope in situ hybridization multiplex version 2 (Advanced Cell Diagnostics; Cat 323100) was conducted on human spinal cord sections using the fresh frozen protocol as described by ACD (acdbio; manual # 323100‐USM with rev date: 02272019). Hydrogen peroxide (ACD; Cat 322381) was applied to each section until fully covered and incubated for 10 min at room temperature. The slides were then washed in distilled water and then incubated one at a time in protease III reagent (ACD; Cat 322381) for 10 s at room temperature. Slides were then washed briefly in 1x phosphate‐buffered saline (PBS, pH 7.4) at room temperature.

A positive and negative control was run on single sections from each spinal cord for every RNAscope experiment. Due to the high lipofuscin content and high autofluorescence in human nervous tissues, only two mRNAs of interest (*SCN9A* [ACD; Cat 562251] with *TACR1* [ACD; Cat 310701] and *SCN9A* with *GPR83* [ACD; Cat 1082151]) were stained at a time (in the Cy3 and Cy5 channels), and a third channel (488 channel) was left unstained to reveal background signal. The only exception to this was with the positive and negative control probes, as these contain a premixed combination of three mRNA probes. The positive control probe cocktail (ACD; Cat 320861) contains probes for high‐, medium‐, and low‐expressing mRNAs that are present in all cells (ubiquitin C > peptidyl‐prolyl cis–trans isomerase B > DNA‐directed RNA polymerase II subunit RPB1) and allows us to gauge tissue quality and experimental conditions. All tissues showed a robust signal for all three positive control probes. A negative control probe cocktail (ACD; Cat 320871) against the bacterial DapB gene was used to check the background label due to the experimental assay.

Each slide was then placed in a prewarmed humidity control tray (ACD; Cat 321710) containing dampened filter paper (ThermoFisher Scientific; Cat 84784), and the probes were pipetted onto each section until fully covered. This was performed one slide at a time to avoid liquid evaporation and section drying. The humidity control tray was placed in a HybEZ oven (ACD; Cat 321710) for 2 h at 40°C. Following probe incubation, the slides were washed two times in 1x RNAscope wash buffer (ACD; Cat 310091) and then placed in 5x SSC buffer (Sigma; Cat S6639) overnight at room temperature.

The following morning, the slides were washed two times in 1x RNAscope wash buffer (ACD; Cat 310091) and placed in the 40°C oven for 30 min after submersion in AMP‐1 reagent. Washes and amplification were repeated using AMP‐2 and AMP‐3 reagents with a 30‐ and 15‐min incubation period, respectively. HRP‐C1 reagent was applied to all sections and then incubated in the oven at 40°C for 15 min. The slides were then washed in 1x RNAscope wash buffer (ACD; Cat 310091). TSA Plus Akoya Dyes in Fluorescin, Cy‐3, and Cy‐5 (Akoya; NEL741001KT, NEL744001KT, NEL745001KT) were prepared at 1:1000 in TSA buffer (ACD; Cat 322809). The Akoya dye assigned to the Channel 1 probe was applied to each section until fully covered and incubated for 30 min in the 40°C oven. The slides were washed and then covered in HRP blocker (ACD; Cat 323110) for 15 min at 40°C. The slides were washed again, and then the same steps were repeated using HRP‐C2 and HRP‐C3 reagents with their assigned Akoya dye. DAPI (Cayman Chemical; Cat 14285) was prepared at 1:5000 in RNAscope wash buffer and was applied to each section for 1 min at room temperature. The slides were then washed in RNAscope wash buffer, air‐dried, and cover‐slipped (Globe Scientific; Cat 1415‐15) with Prolong Gold Antifade mounting medium (Fisher Scientific; Cat P36930).

### 2.3 RNAscope In Situ Hybridization Imaging and Analysis

One side of each spinal cord section (three sections per donor, three to four donors total) was imaged mosaically on an FV4000 confocal microscope (Evident Scientific), which has autofocus and autostitching capabilities. All mosaic images were autostitched 20x confocal *xy* images and were not projected *z* stacks. The acquisition parameters were set based on guidelines for the FV4000 provided by Evident Scientific. The raw image files were analyzed in CellSens (Olympus; v1.18). The True black lipofuscin quencher (used in immunofluorescence) is not compatible with RNAscope. Large globular structures and/or signal that auto‐fluoresced in all channels and were visible in the unstained 488 channel were considered to be background lipofuscin and were not considered to be mRNA signal. Aside from adjusting brightness/contrast, we performed no digital image processing to subtract the background. Images of sections that were tilted were rotated so that laminar boundaries were more distinguishable. The DAPI co‐stain was useful in defining the white versus gray matter boundaries and in visualizing the substantia gelatinosa. The spinal level of each section was determined by comparing the images with a human spinal cord atlas (Sengul et al. 2013) and is indicated in Table 1.

Laminar boundaries in the human spinal cord atlas (Sengul et al. 2013) were measured with a ruler and converted to micrometers using the atlas's scalebar. The mosaic images were opened in CellSens, and micrometer guidelines were drawn using the polyline tool for each laminar boundary determined in the atlas. The only boundary exceptions made were with the lamina II boundaries, which were clearly defined by the outline of the substantia gelatinosa but did not always completely align with the atlas. The perimeter of each lamina was traced using the closed polygon tool. The number of DAPI+ nuclei that were positive for each mRNA target was counted within each lamina. The total number of nuclei that were positive for either *SCN9A*, *TACR1*, and/or *GPR83* is shown in Table 2. It is important to note that DAPI weakly stains the nuclei of larger‐sized neurons, an observation we have frequently noted in our many experiments working with human nervous tissues. In the circumstances where we saw robust mRNA signal but faint or diminished nuclei signal, the image was brightened to confirm that a nucleus was present. For purposes of figure display, this may not be obvious. Graphs were generated using GraphPad Prism version 8.4.3 (GraphPad Software, Inc., San Diego, CA, USA).

**TABLE 2:** Number of positive *SCN9A*, *TACR1*, and/or *GPR83* nuclei within the dorsal horn per donor.

Donor ID	Experiment	SCN9A+ cells	Experiment	SCN9A+ cells
UTD‐DN0042	*SCN9A*/*TACR1*	1007	*SCN9A*/*GPR83*	1596
UTD‐DN0087	*SCN9A*/*TACR1*	2468	*SCN9A*/*GPR83*	1786
UTD‐DN0077	*SCN9A*/*TACR1*	510	*SCN9A*/*GPR83*	531
UTD‐DN0044	*SCN9A*/*TACR1*	1452	*SCN9A*/*GPR83*	1153

### 2.4 Immunofluorescence and Imaging

Human spinal cords were sectioned at 20 µm onto SuperFrost Plus charged slides (Fisher Scientific; Cat 12‐550‐15). Sections were only briefly thawed in order to adhere to the slide but were immediately returned to the −20°C cryostat chamber until completion of sectioning. Three sections from each donor were stained in each experiment, and three to four donors were used in each experiment. A negative control section from each spinal cord was exposed to all reagents except for the primary antibody.

Slides were removed from the cryostat and immediately transferred to cold 10% formalin (4°C; pH 7.4) for 15 min. The tissues were then dehydrated in 50% ethanol (5 min), 70% ethanol (5 min), 100% ethanol (5 min), and 100% ethanol (5 min) at room temperature. The slides were air‐dried briefly, and then boundaries were drawn around each section using a hydrophobic pen (ImmEdge PAP pen, Vector Labs). When hydrophobic boundaries had dried, the slides were submerged in blocking buffer (10% Normal Goat Serum, 0.3% Triton‐X 100 in 1x PBS) for 1 h at room temperature. Slides were then rinsed in 1x PBS, placed in a light‐protected, humidity‐controlled tray, and incubated in primary antibodies diluted in blocking buffer overnight at 4°C. A list of the antibodies used can be found in Table 3. The Na_V_1.7 mouse monoclonal antibody has been knockout validated using immunocytochemistry on mouse cultured DRG neurons and using IHC on the rat brain (Grubinska et al. 2019). We have also shown that this antibody robustly stains human DRG and shows a specific and similar expression pattern to its mRNA in human DRG (S. Shiers et al. 2020).

**TABLE 3:** Antibodies used for immunofluorescence.

Antibody	Vendor	Catalog # or Clone #	RRID	Dilution/conc.
Mouse‐anti‐ Na_V_1.7	NeuroMab	N68/6	AB_2184355	2 µg/mL
Rabbit‐anti‐NeuN	Cell Signaling	24307S	AB_2651140	1:500
Rabbit‐anti‐CGRP	ImmunoStar	24112	AB_572217	1:1000
Mouse‐anti‐Bassoon	Enzo Life Sci	SAP7F407	AB_11181058	2 µg/mL
Mouse‐anti‐Ankyrin G	NeuroMab	N106/36	AB_10673030	2 µg/mL
Chicken‐anti‐MAP2	Novus Bio	NB300‐213	AB_2138178	2 µg/mL
Goat‐anti‐rabbit H&L 647	ThermoFisher	A21245	AB_2535813	1:2000
Goat‐anti‐chicken H&L 647	ThermoFisher	A21449	AB_2535866	1:2000
Goat‐anti‐mouse IgG2a 555	ThermoFisher	A21137	AB_2535776	1:2000
Goat‐anti‐mouse IgG2a 488	ThermoFisher	AB21131	AB_2535771	1:2000
Goat‐anti‐mouse IgG1 488	ThermoFisher	A21121	AB_2535764	1:2000
Goat‐anti‐mouse IgG1 555	ThermoFisher	A21127	AB_2535769	1:2000

The next day, slides were washed in 1x PBS and then incubated in their respective secondary antibody diluted at 1:2000 with DAPI (1:5000; Cayman Chemical; Cat 14285) in blocking buffer for 1 h at room temperature. The sections were washed in 1x PBS and then covered with True Black (20% diluted in 70% Ethanol), a blocker of lipofuscin, for 1 min. Sections were then washed in water, air‐dried, and coverslipped with Prolong Gold Antifade reagent (Cat P36930, Fisher Scientific).

One side of each spinal cord section (three sections per donor) was imaged mosaically on an FV3000 or FV4000 confocal microscope (Evident Scientific), which has autofocus and autostitching capabilities. All mosaic images were autostitched 20x confocal *xy* images and were not projected *z* stacks. Additionally, 10x, 20x, and 40x *xy* images were also acquired on either a FV3000 or FV4000 confocal microscope. The acquisition parameters were set based on guidelines for each microscope provided by Evident Scientific. The raw image files were visualized in CellSens (Olympus; v1.18). Images were brightened and contrasted in CellSens, and the negative control was equally adjusted.

## 3 Results

### 3.1 *SCN9A* mRNA Is Expressed in *TACR1*+ Putative Projection Neurons in the Human Spinal Dorsal Horn

**FIGURE 1:**
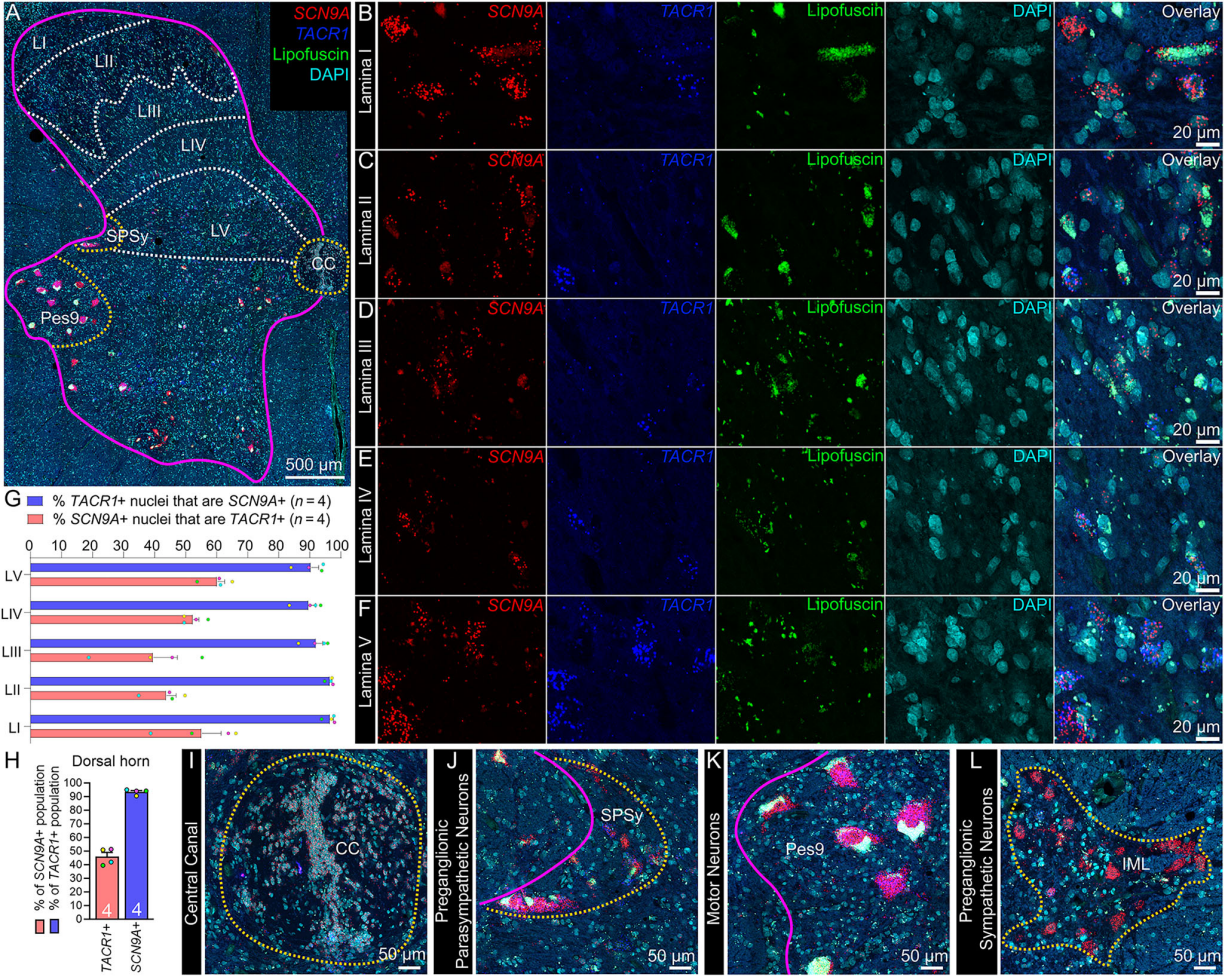
Distribution of *SCN9A* (Na_V_1.7) and *TACR1* (NK1R) mRNAs in the human spinal dorsal horn using RNAscope. (A) Mosaic image of a human spinal cord (lumbar 5/sacral 1) labeled with RNAscope in situ hybridization for *SCN9A* (red) and *TACR1* (green) mRNAs and co‐stained with DAPI (cyan). The 488 channel was left unstained (green) to reveal background autofluorescence and lipofuscin, which is present in all human neurons. Magenta line outlines gray matter. Higher magnification images for each channel are shown for (B) lamina I, (C) lamina II, (D) lamina III, (E) lamina IV, and (F) lamina V. (G) Percentage of *SCN9A*+ nuclei that coexpressed *TACR1* (red bar) and percentage of *TACR1*+ nuclei that coexpressed *SCN9A* (blue bar) for each lamina (LI–LV). (H) Percentage of *TACR1*+ nuclei that were copositive for *SCN9A* (blue bar) and percentage of *SCN9A*+ nuclei that were copositive for *TACR1* (red bar) for the entire dorsal horn (combined data of H). (I) S*CN9A* mRNA was also detected in (I) nuclei lining the central canal, likely ependymal cells, (J) preganglionic parasympathetic neurons in the sacral parasympathetic nucleus (SPSy), (K) motor neurons in the ventral horn, particularly Pes9, which contains motor neurons associated with the feet, and (L) preganglionic sympathetic neurons in the intermediolateral column (IML) from a lumbar 1 spinal cord section. LI–LV, lamina I–V; SPSy, sacral parasympathetic nucleus; Pes9, motor neurons of the foot; CC, central canal. Sample size: *n* = 4. Scale bars: A = 500 µm; B–F = 20 µm; I–L = 50 µm.

The substance P receptor (protein: Nk1r, mRNA: *Tacr1*) labels a subset of projection neurons in the rodent spinal dorsal horn that transmit pain and itch information to the parabrachial nucleus in the brainstem (Blomqvist and Mackerlova 1995; Carstens et al. 2010; Mantyh et al. 1997). These neurons are localized to the superficial laminae, primarily lamina I (Carstens et al. 2010; Mantyh et al. 1997), but they are also found in the deep dorsal horn, mostly in lamina V (Brown et al. 1995). It is important to note that the molecular identity of human projection neurons remains unknown, and as such, we refer to the large *TACR1*/Nk1r‐ and *GPR83*‐expressing neurons in lamina I and V of the dorsal horn as *putative* projection neurons. Although Nk1r/*Tacr1* is not exclusive to the projection neuron population (Russ et al. 2021), it is commonly used to identify dorsal horn projection neurons (Marshall et al. 1996). We assessed the distribution of *SCN9A* (Na_V_1.7) and *TACR1* (Nk1r) mRNA in the human spinal dorsal horn using RNAscope. We found that *SCN9A* and *TACR1* mRNAs were detected in cells throughout lamina I–V in the human spinal dorsal horn (Figure 1A–F). In the dorsal horn (laminae I–V), virtually all of the *TACR1*+ cells were *SCN9A*+ (92%), but only 45% of *SCN9A*+ cells were *TACR1*+ (Figure 1G,H). *SCN9A* mRNA was also detected in ependymal cells near the central canal (Figure 1I), preganglionic parasympathetic neurons in the sacral parasympathetic nucleus (Figure 1J), motor neurons in the ventral horn (Figure 1K), and preganglionic sympathetic neurons in the intermediolateral column (Figure 1L).

### 3.2 *SCN9A* mRNA Is Expressed in *GPR83*+ Putative Projection Neurons in the Human Spinal Dorsal Horn

**FIGURE 2:**
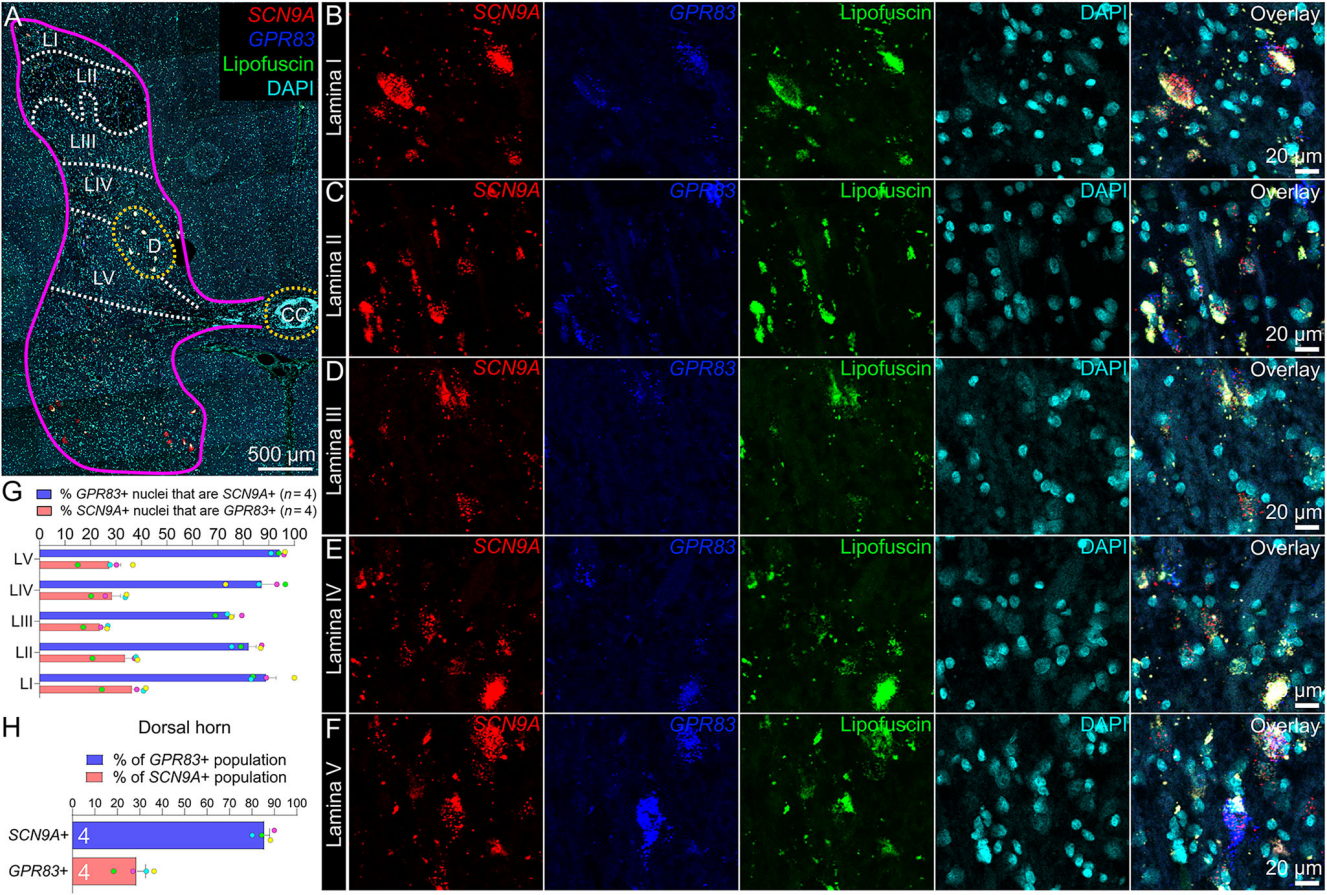
Distribution of *SCN9A* (Na_V_1.7) and *GPR83* mRNAs in the human spinal dorsal horn using RNAscope. (A) Mosaic image of a human spinal cord section (lumbar 3) labeled with RNAscope in situ hybridization for *SCN9A* (red) and *GPR83* (green) mRNAs and co‐stained with DAPI (cyan). The 488 channel was left unstained (green) to reveal background autofluorescence and lipofuscin, which is present in all human neurons. Magenta line outlines gray matter. Higher magnification images for each channel are shown for (B) lamina I, (C) lamina II, (D) lamina III, (E) lamina IV, and (F) lamina V. (G) Percentage of *SCN9A*+ nuclei in that coexpressed *GPR83* (red bar) and percentage of *GPR83*+ nuclei that coexpressed *SCN9A* (blue bar) for each lamina (LI–LV). (H) Percentage of *GPR83*+ nuclei that were copositive for *SCN9A* (blue bar) and percentage of *SCN9A*+ nuclei that were copositive for *GPR83* (red bar) for the entire dorsal horn (combined data of H). LI–LV, lamina I–V; D, dorsal nucleus; CC, central canal. Sample size: *n* = 4. Scale bars: A = 500 µm; B–F = 20 µm.

**FIGURE 3:**
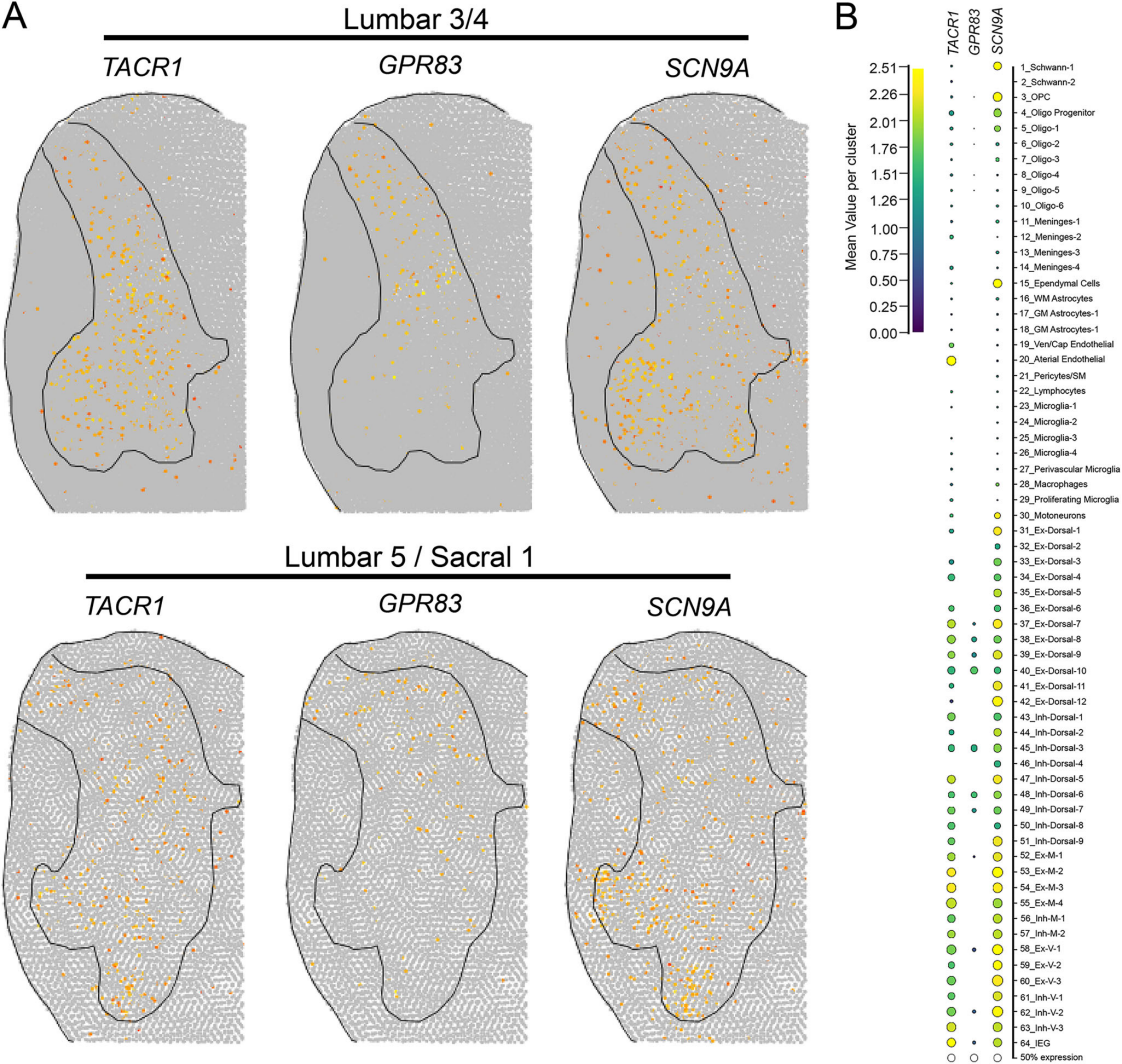
Spatial and single‐nuclear RNA‐sequencing detection of *SCN9A* and *TACR1* in the human spinal dorsal horn. (A) Normalized spatial transcriptomic gene expression for *SCN9A*, *GPR83*, and *TACR1* per barcoded spot on aggregated human lumbar spinal cord sections (Yadav et al. 2023). Solid lines mark gray matter boundaries. (B) Dot plot showing the average gene expression for *SCN9A*, *GPR83*, and *TACR1* for each human spinal cord cluster identified using single‐nucleus RNA sequencing. Data obtained from https://vmenon.shinyapps.io/humanspinalcord/.

The G protein‐coupled receptor 83 (GPR83) is expressed by a second projection neuron population found in lamina I–II of the rodent spinal dorsal horn. Both the GPR83 and Tacr1 projection neuron populations show little overlap (∼20% overlap), terminate in different subnuclei of the parabrachial nucleus, and, together, comprise ∼88% of all ALS projection neurons (Choi et al. 2020). However, a recent report with a new transgenic line recapitulates older findings (Cameron et al. 2015), which indicate that *Tacr1* is expressed in 90% of all ALS projection neurons (Ma et al. 2025), suggesting that these populations may not be as distinct as previously reported. To further investigate the expression of *SCN9A* in projection neurons, we conducted RNAscope in situ hybridization in combination with *GPR83* (Figure 2A). *SCN9A* and *GPR83* were detected in cells throughout lamina I–V (Figure 2B–F). *SCN9A* was coexpressed in the majority of *GPR83*‐expressing cells across lamina I–V (Figure 2G), with aggregate data for the region indicating that 89% of all *GPR83*‐expressing cells coexpressed *SCN9A* mRNA (Figure 2H). This distribution closely resembles recently published human spinal cord spatial transcriptomic data (Yadav et al. 2023), in which *SCN9A*, *TACR1*, and *GPR83* mRNAs were found throughout the dorsal and ventral horns (Figure 3A). *SCN9A* was also detected in a broad population of excitatory and inhibitory dorsal horn neurons using single‐nucleus RNA sequencing of human spinal cord (Yadav et al. 2023), many of which co‐expressed *TACR1* and/or *GPR83* (Figure 3B).

### 3.3 Na_V_1.7 Protein is Localized Presynaptically in the Human Spinal Cord

**FIGURE 4:**
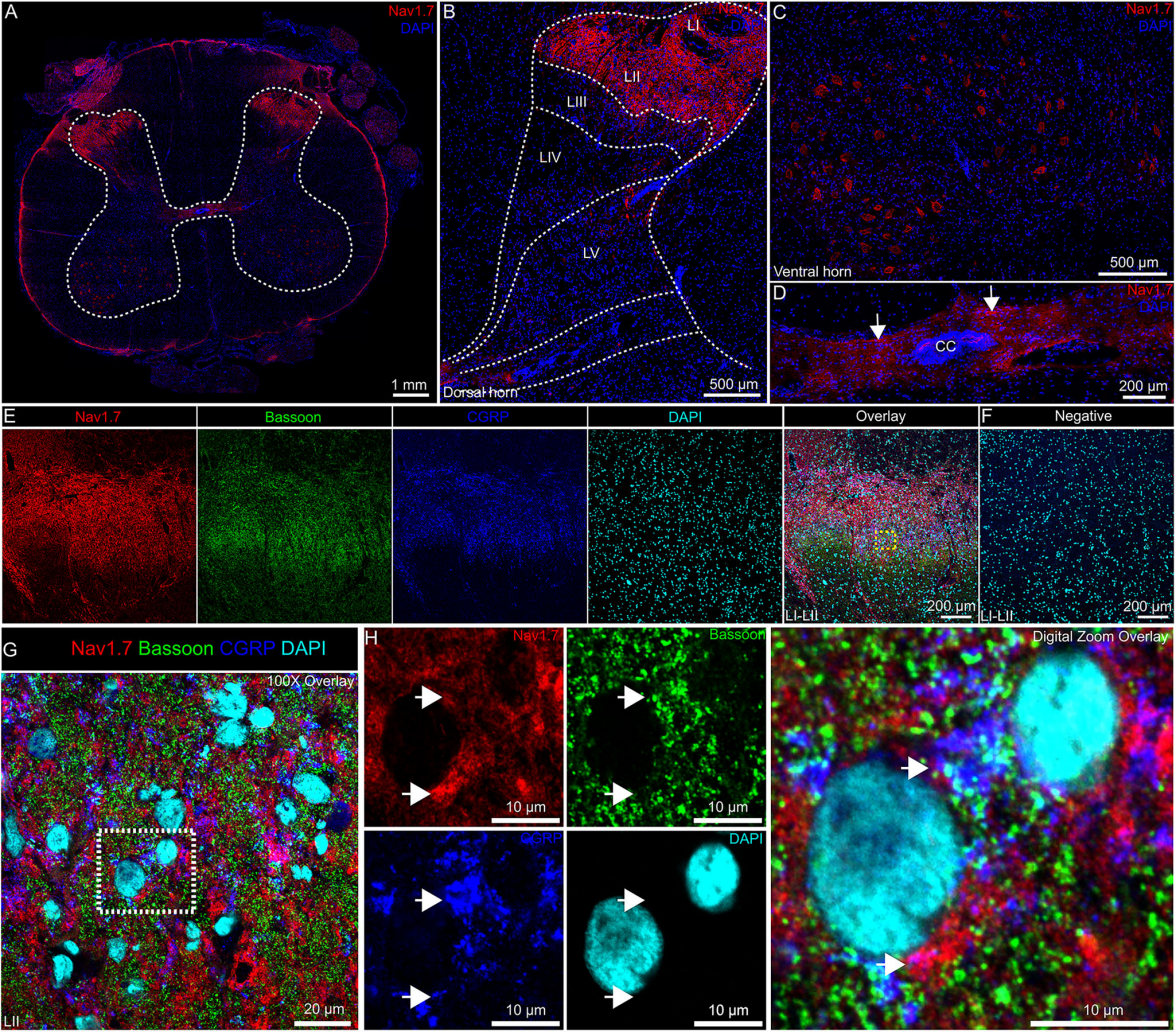
Na_V_1.7 protein expression in the human lumbar spinal cord. Mosaic image of Na_V_1.7 (red) protein staining in the human lumbar spinal cord (lumbar 5), co‐stained with DAPI (blue). White outline demarcates the gray matter. (B) Na_V_1.7 protein gave an axonal and neuropil (synaptic) pattern in the spinal dorsal horn, mostly localized to lamina I–II (LI–LII). (C) Nav1.7 protein was also detected in the cytoplasm of large motor neurons in the ventral horn and (D) in axons in the anterior commissure (white arrows), as well as the cytoplasm of ependymal cells (cells outlining the central canal). (E) Representative 10x magnification confocal image of Na_V_1.7 protein (red) co‐stained with the nociceptive presynaptic marker CGRP (blue), the presynaptic active zone marker Bassoon (green), and DAPI (cyan) in LI–LII of the spinal dorsal horn. (F) Overlay image (488, 555, 647, DAPI) of LI–LII in the negative control that was exposed to all of the same reagents except primary antibody and imaged and adjusted to the same settings as shown in E. (G) A higher magnification (100x) confocal view of the area outlined in yellow in the overlay image in C. (F) A digitally magnified view of the area outlined in white in E showing Na_V_1.7 signal colocalized with CGRP and/or Bassoon (white arrows) or in close proximity to these proteins. LI–LV, lamina I–V; CC, central canal. Sample size: *n* = 4. Scale bars: A = 1 mm; B–C = 500 µm; D–F = 200 µm; G = 20 µm; H = 10 µm.

Na_V_1.7 protein staining in the human spinal cord was robustly found in the dorsal rootlet and lamina I and II, as we have previously reported (S. I. Shiers et al. 2021) (Figure 4A,B). Na_V_1.7 staining gave an axonal and synaptic (neuropil) pattern throughout lamina I–II (Figure 4B). Na_V_1.7 was detected in the cytoplasm of motor neurons in the ventral horn (Figure 4C), axons in the anterior commissure (Figure 4D), and ependymal cells lining the central canal, corroborating its mRNA expression in those cells (Figure 4D).

When colabeled with CGRP, a neuropeptide that we have shown is expressed in all human nociceptors (S. Shiers et al. 2020; S. I. Shiers et al. 2021), and the presynaptic active‐zone protein, Bassoon (Figure 4E), the majority of the Na_V_1.7 signal appeared to colocalize with, or to be in close proximity to, these presynaptic markers above background signal (Figure 4F). However, higher magnification imaging revealed that not all of the Na_V_1.7 signal in the superficial laminae appeared to be localized with these presynaptic proteins (Figure 4G,H), which could be indicative of differences in the subcellular localization of these proteins within the presynaptic compartment, traveling DRG primary afferents that have not yet met their termination point within the dorsal horn, or even dendritic or axonal localization of Na_V_1.7 in resident dorsal horn neurons.

Indeed, Na_V_1.7 was also detected in axons in deeper lamina (LIII–LV), with the highest abundance in lamina V (Figure 4D) and also in axons entering the anterior commissure (near lamina X and the central canal), where dorsal horn projection neurons cross the midline to enter the contralateral spinal thalamic tract. As tracing studies in cats and macaques have demonstrated that neurons projecting to the spinal thalamic tract originate in lamina I and lamina V, it is possible that the non‐presynaptic signal in the superficial lamina as well as the axonal signal in the deep lamina and anterior commissure could represent Na_V_1.7 protein expressed by projection neurons (Craig 2006; Jones et al. 1987; Willis et al. 1979), a hypothesis we explored with additional experiments.

### 3.4 Evidence for Postsynaptic Na_V_1.7 Protein in the Human Spinal Cord

In an effort to identify postsynaptic Na_V_1.7 immunoreactivity in the spinal dorsal horn, we co‐stained Na_V_1.7 with a variety of neuronal subcellular protein markers. While Nav1.7 is localized within the cytoplasm and membrane of the cell body of sensory neurons, as well as their peripheral and central axons and terminal synapses, its expression in the CNS may differ. Other VGSCs are known to be expressed in different cellular compartments in the CNS, such as the synapse, dendrites, axon initial segment (AIS), nodes of Ranvier, and axons of neurons throughout the spinal cord and brain (Hanson et al. 2004; Schaller and Caldwell 2003).

**FIGURE 5:**
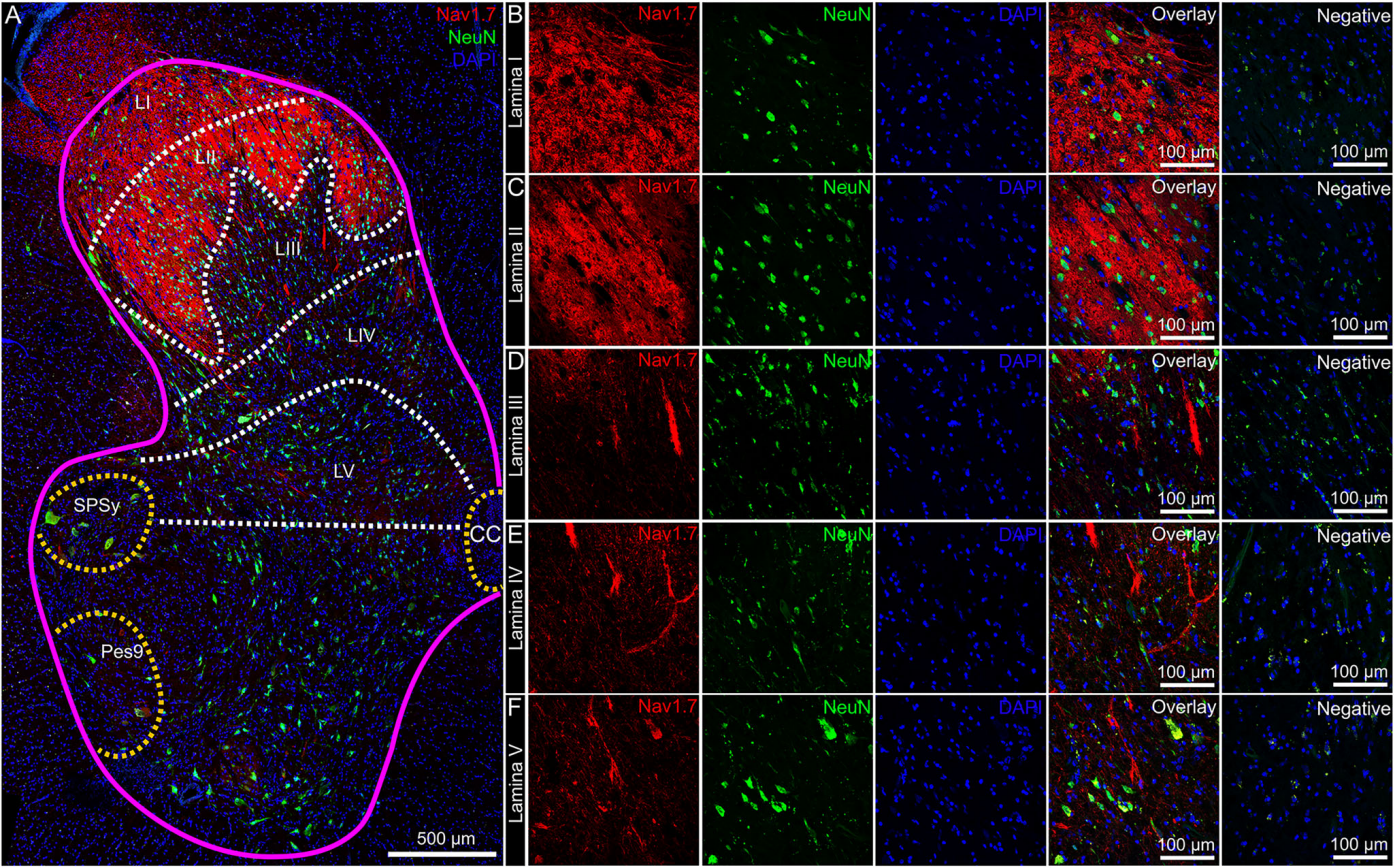
Investigation of Na_V_1.7 protein in the soma of resident neurons in the dorsal horn. (A) Mosaic image of a human spinal cord section (lumbar 5–sacral 1) immunolabeled with Na_V_1.7 (red), NeuN (green, soma and nucleus of neurons), and DAPI (blue, nuclei). Magenta line outlines the gray matter. Representative 40x images of Na_V_1.7 (red), NeuN (green), and DAPI (blue) in (B) lamina I, (C) lamina II, (D) lamina III, (E) lamina IV, and (F) lamina V and their corresponding negative control that was exposed to all of the same reagents except primary antibody and imaged and adjusted with the same settings of each subregion. Na_V_1.7 was not detected in the soma of any of the dorsal horn (lamina I–V) neurons. However, it was detected in the cell bodies of motor neurons and in preganglionic parasympathetic neurons. LI–LV, lamina I–V; SPSy, sacral parasympathetic nucleus; Pes9, motor neurons of the foot; CC, central canal. Sample size: *n* = 4. Scale bars: A = 500 µm; B–F = 100 µm.

**FIGURE 6:**
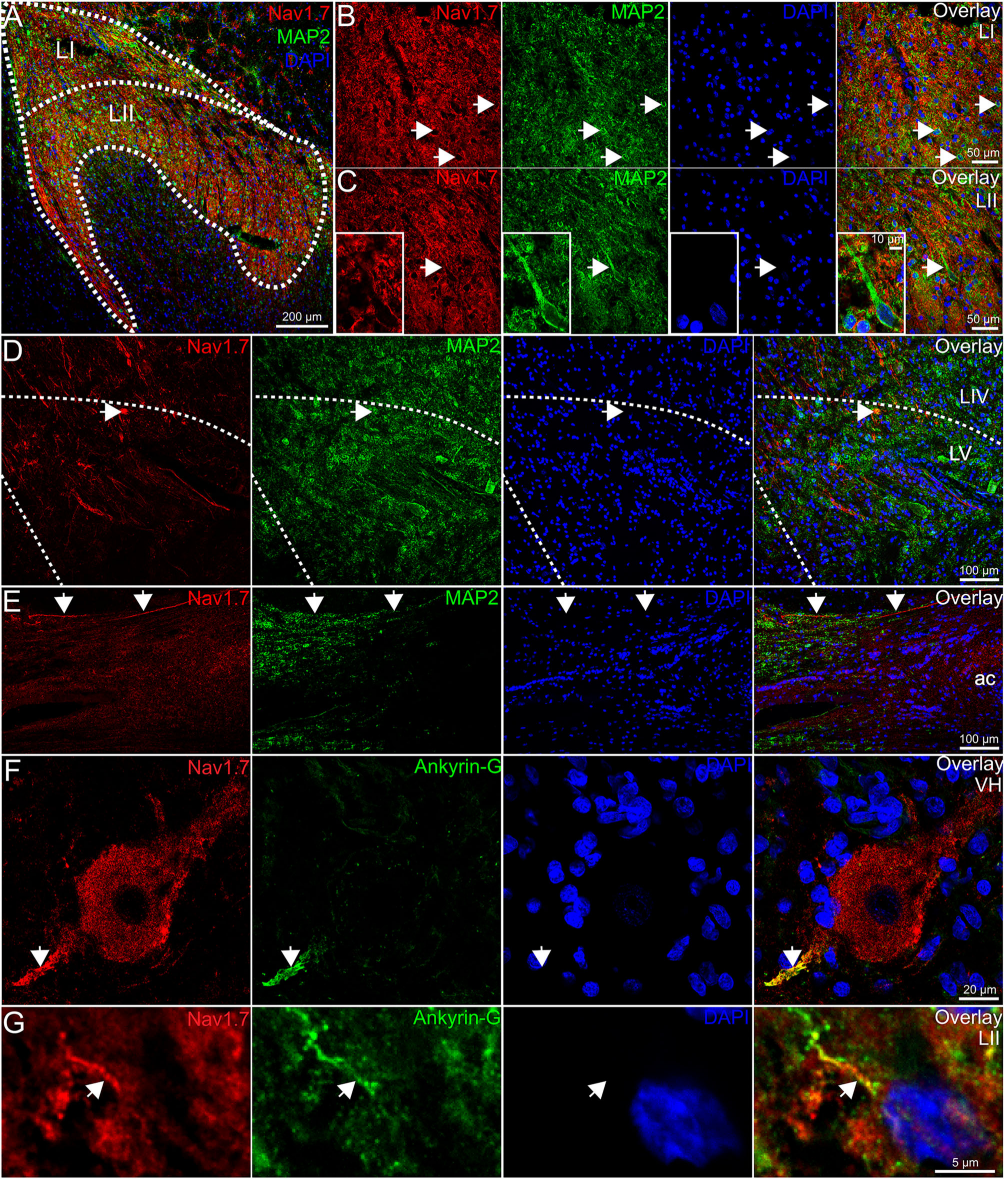
Evidence for postsynaptic Na_V_1.7 expression in the human spinal cord. (A) Representative 10x image of Na_V_1.7 (red), MAP2 (green), and DAPI (blue) staining in the human lumbar dorsal horn. The white outlines lamina I and lamina II. Representative 40x images of Na_V_1.7 (red), MAP2 (green), and DAPI (blue) in (B) lamina I and (C) lamina II. White arrows point to the MAP2 signal that is localized around resident neurons (appears to be the plasma membrane) but is absent of Na_V_1.7 signal. The image inset in panel C shows a 100x image of a large, LII neuron with a large apical dendrite that is devoid of Na_V_1.7 signal. (D) Representative 20x image of Na_V_1.7‐positive axonal fibers in the deeper lamina around LIV–LV. The white arrow points to Na_V_1.7 and MAP2 copositive signal (yellow in overlay) that does not have a nucleus and is not a cell body. (E) Representative 20x image of Na_V_1.7 staining in the anterior commissure (ac), where intensely labeled Na_V_1.7‐positive axons are highlighted (white arrow). (F) A 100x image of Na_V_1.7 (red), Ankyrin‐G (green), and DAPI (blue) staining in a motor neuron in the ventral horn. (G) A cropped, zoomed‐in image of Na_V_1.7 (red), Ankyrin‐G (green), and DAPI (blue) signal in a lamina II dorsal horn neuron. LI–LV, lamina I–V; ac, anterior commissure. Sample size: *n* = 3–4. Scale bars: A = 200 µm; B and C = 50 µm; D and E = 100 µm; F = 20 µm; G = 5 µm.

First, we assessed Na_V_1.7 in combination with the neuronal soma and nuclear marker, NeuN (Figure 5A), to determine if Na_V_1.7 was detected in the soma of resident dorsal horn neurons. However, we found no convincing evidence that Nav1.7 was expressed in the cell bodies of neurons in lamina I–V (Figure 5B–F). Next, we assessed if Nav1.7 was coexpressed with the cytoskeletal marker, MAP2, which is primarily localized to dendrites (Figure 6A). We assessed the dorsal horn from three organ donors but did not find evidence for Na_V_1.7 signal localized to the dendritic compartment of resident dorsal horn neurons (Figure 6B–D). In the anterior commissure, we again observed intensely labeled Na_V_1.7 axonal fibers crossing to the other side of the spinal cord (Figure 6E), but we could not determine what neurons these axons originated from; however, it is likely they are projection neurons, as this is a major hub for spinal neurotransmission.

Interestingly, we noted that Na_V_1.7 was localized not only to the soma of motor neurons but also to what appeared to be their AIS. Recent work with Na_V_1.7 in the rodent DRG found that sensory neurons contain an AIS, that it is enriched with Na_V_1.7, and that its localization there is critical for spontaneous activity in neuropathic pain (Nascimento et al. 2022). Other VGSCs are also known to be enriched in the AIS in the CNS (Leterrier 2018). To confirm our observation in motor neurons, we co‐stained Na_V_1.7 with the AIS marker, Ankyrin‐G, and found that Na_V_1.7 colocalized with Ankyrin‐G at the AIS of motor neurons in the human ventral horn (Figure 6F). In the dorsal horn, we also found evidence for Na_V_1.7 localized to the AIS of resident neurons (Figure 6G), but this staining was sparse and was not detected in all neurons. It is important to note that postsynaptic Na_V_1.7 signal within LI–LII was difficult to detect due to the intense presynaptic neuropil labeling throughout the region. Na_V_1.7 expression within resident spinal dorsal horn neurons may have been low, or below the level of detection. However, increasing the imaging parameters to capture this signal would result in oversaturation of the presynaptic label and make it so that cellular structures were no longer distinguishable. As such, we kept the imaging parameters low so that the anatomy could be visualized through the neuropil staining. This may be indicative of why the detection of Na_V_1.7 postsynaptic label was lower than expected. However, given that we observed robust Na_V_1.7 mRNA expression in most *TACR1*+ and *GPR83*+ neurons and found evidence of Na_V_1.7 localization at AISs in dorsal and ventral horn neurons, our data support that when the mRNA is translated, it is localized to the membrane of intrinsic dorsal horn axons. This is further supported by Na_V_1.7 axonal labeling in the anterior commissure.

## 4 Discussion

In the present study, we found evidence for the presence of Na_V_1.7 expression in intrinsic neurons of the human spinal cord, including in the dorsal and ventral horns. First, we identified that *SCN9A* (protein: Na_V_1.7) mRNA was detected in ∼90% of all *GPR83+* and *TACR1*+ (protein: Nk1r) neurons, which are known markers of projection neurons in rodents (Cameron et al. 2015; Carstens et al. 2010; Choi et al. 2020; Ma et al. 2025; Mantyh et al. 1997). In rodents, the Nk1r and GPR83 are expressed by a subset of large‐diameter projection neurons that send projections to higher brain regions like the parabrachial nucleus, and are found predominantly in lamina I and in the deeper dorsal horn around lamina V (Brown et al. 1995; Choi et al. 2020; Marshall et al. 1996; Todd et al. 2000). It is important to note that not all rodent projection neurons are *Tacr1*+ (Nk1r+), and interneurons as well as motor neurons also express this gene (Russ et al. 2021). Single‐nucleus RNA sequencing recapitulates this expression pattern as *SCN9A*, *GPR83*, and *TACR1* mRNAs were co‐expressed in a wide range of human spinal neuronal populations, including motor neurons (Yadav et al. 2023). The high co‐expression of *SCN9A* with known rodent projection neuron markers in LI–LII and LV supports that a subset of the *SCN9A*, *GPR83*, and *TACR1* co‐expressing neurons are likely projection neurons.

Another study detected *Scn9a* mRNA in a variety of Tacr1 co‐expressing neuron populations in the mouse spinal cord using single‐cell sequencing (Russ et al. 2021). However, only its motor neuron expression could be validated with in situ hybridization (Allen Institute for Brain Science 2022b; Alles et al. 2020). While sensitivity issues could underlie these technical differences, detection of Na_V_1.7 mRNA and protein in rodent spinal dorsal horn neurons has also been inconclusive, likely due to its unique subcellular localization to the neuronal membrane, which could comprise the soma, dendrites, AIS, nodes of Ranvier, and/or synapse. Indeed, most reports suggest that spinal Na_V_1.7 protein expression is entirely presynaptic due to its robust neuropil staining pattern within lamina I–II (Black et al. 2012; S. I. Shiers et al. 2021). However, immuno‐electron microscopy detected Na_V_1.7 protein localized to dendrites of mouse dorsal horn neurons, but it was hypothesized that these proteins originated in the presynaptic compartment and were transferred to postsynaptic sites through an unknown mechanism (Alles et al. 2020).

While we also detected intense presynaptic Na_V_1.7 labeling in lamina I–II of the human spinal cord, not all of its expression was colocalized with presynaptic markers like CGRP and Bassoon. Na_V_1.7 protein was also localized to axons in the deeper lamina, particularly lamina IV and V, and to axons in the anterior commissure, the white matter tract connecting both sides of the spinal cord, and an important relay for dorsal horn projection neurons transmitting nociceptive information to the contralateral spinothalamic tract (Ku and Morrison 2022). Because axon tracing methods cannot readily be employed in the human spinal cord, we could not identify if these were primary afferents or the axons of intrinsic dorsal horn neurons; however, ascending projections of primary afferents ascend in the dorsal columns and do not cross the midline, so it is exceedingly unlikely that these commissural axons are contributed by sensory neurons.

Interestingly, we did not detect Na_V_1.7 localized to dendrites but instead identified its expression in the AISs of some dorsal horn neurons in lamina I–II and also in motor neurons. Other Nav family members are highly concentrated at the AIS, and their localization there is critical for integration of synaptic currents into action potential generation (Grubb and Burrone 2010; Leterrier 2018). Importantly, neurons are known to increase or decrease the size of their AIS as a means to augment or depress their neuronal excitability in response to changing presynaptic input (Grubb and Burrone 2010; Grubb et al. 2011; Kuba et al. 2010). As sensory neuron hyperexcitability and/or spontaneous activity is a major driver of abnormal nociceptive signaling into the dorsal horn in chronic pain conditions, it is possible that Na_V_1.7 regulation at the AIS in resident dorsal horn neurons is critical for nociceptive processing in the dorsal horn. A recent study found Nav1.7 expression in mouse dorsal horn neurons after spinal cord injury (Fu et al. 2023). This raises the possibility that the presence of mRNA for *SCN9A* in human dorsal horn neurons sets the stage for upregulation of protein expression after injury.

While loss‐of‐function mutations in *SCN9A* result in pain insensitivity in both rodents and humans (Gingras et al. 2014; Grubinska et al. 2019; Shields et al. 2018), gain‐of‐function mutations engineered in mice to match human mutations that cause pain disorders do not recapitulate the human pain phenotype (Chen et al. 2021). One potential explanation for this is that the loss‐of‐function phenotype is dependent upon sensory neuron expression of Na_V_1.7, which is conserved across species, and that the loss of function leads to a conserved loss of action potential generation in nociceptors. On the other hand, the human gain‐of‐function pain phenotype may require both peripheral sensitization and spinal amplification. As there has been little convincing evidence for Nav1.7 expression in intrinsic neurons of the mouse spinal cord in the absence of injury, one hypothesis is that spinal amplification of nociceptive signals does not occur in mice but may occur in humans with gain‐of‐function mutations, given the broad expression of *SCN9A* in dorsal horn neurons, including putative projection neurons. If this is the case, gain‐of‐function mutations in *SCN9A* could lead to increased excitability of projection neurons, which would be further amplified by increased nociceptive input from hyperexcitable nociceptors in the periphery. However, this mechanistic hypothesis may not be as straightforward as this because *SCN9A* mRNA was found in many neurons in the dorsal horn, including inhibitory interneurons, by single‐nucleus sequencing (Yadav et al. 2023). Nevertheless, dysregulated circuit dynamics in the dorsal horn caused by gain‐of‐function mutations in Nav1.7 potentially explain the difference in pain phenotype between humans and rodents.

In summary, we offer several compelling pieces of evidence to support the existence of Na_V_1.7 mRNA and protein expression by intrinsic neurons of the human spinal dorsal horn: (1) *SCN9A* mRNA is expressed by human resident dorsal horn neurons detected by both RNAscope in situ hybridization, spatial sequencing, and single‐nucleus sequencing; (2) ∼90% of *GPR83*+ and *TACR1*+ human resident dorsal horn neurons express *SCN9A*, a subset of which are likely projection neurons; (3) many *SCN9A*+ and *GPR83* or *TACR1* co‐expressing neurons were large diameter and were most abundant in laminar regions (lamina I and lamina V) that are known to be enriched with projection neurons; (4) not all Na_V_1.7 protein signal was limited to the presynaptic compartment as demonstrated with co‐labeling with CGRP and Bassoon; (5) Na_V_1.7 was detected in the AIS of some resident dorsal horn neurons; and (6) Na_V_1.7+ axons were detected in the anterior commissure.

## Funding

This work was supported by NIH grant U19NS130608 and a research grant from Grünenthal.

## Conflicts of Interest

The authors declare no conflicts of interest. J.L.K. and C.L. are employees of Eli Lilly. S.H. is an employee of Grünenthal GmbH.

## Data Availability

The data that support the findings of this study are available from the corresponding author upon request.
